# Common Bacterium Induces Histamine Production in Neutrophils

**DOI:** 10.1289/ehp.120-a190

**Published:** 2012-05-01

**Authors:** Carol Potera

**Affiliations:** Carol Potera, based in Montana, has written for *EHP* since 1996. She also writes for *Microbe*, *Genetic Engineering News*, and the *American Journal of Nursing*.

Airway inflammation and constriction associated with the release of histamine is a hallmark of asthma and allergies. Mast cells are considered the main producers of histamine.^1^ But new research indicates that the common environmental bacterium *Pseudomonas aeruginosa* can induce neutrophils, a type of white blood cell, to produce and release very high levels of histamine as well.^2^

Acute bacterial and viral infections often trigger serious asthma attacks that result in hospitalization, and many of these patients have elevated levels of neutrophils in their airways.^3^ “We suspected that neutrophils may [produce] mediators that worsen airway constriction,” says George Caughey, chief of the Pulmonary and Critical Care Medicine Section at the Veterans Affairs Medical Center and a professor at the University of California, San Francisco, who led the study.

A few years ago, Caughey’s laboratory studied mice infected with the bacterium *Mycoplasma pulmonis* and found that their neutrophils released histamine.^2^ The researchers speculated that the more common and more virulent *P. aeruginosa*—which, like *M. pulmonis*, is associated with chronic airway infections—also may trigger histamine production by neutrophils.

To explore the connection, the researchers infected mouse neutrophils with two clinical laboratory strains of *P. aeruginosa*. Strain PA103 quickly killed the neutrophils, and no extra histamine was produced. The other strain, PAO1, provoked neutrophils to increase histamine production by as much as six times. However, mast cells treated with PAO1 produced no extra histamine.

When mice were infected with PAO1, they developed bronchitis and pneumonia, and lung histamine levels rose by about three times. The histamine surge was tracked to histidine decarboxylase, a key enzyme for histamine synthesis—neutrophils made more of the enzyme when infected with *P. aeruginosa*.^2^

**Figure:**
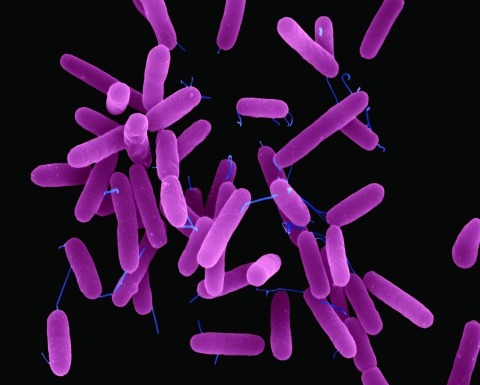
Pseudomonas aeruginosa © Dennis Kunkel/Microscopy, Inc.

The underlying causes of asthma and allergies are not well understood, but the findings suggest that *P. aeruginosa* may trigger inflammation via neutrophils. Patients with chronic airway diseases often experience cycles of bacterial infections followed by inflammation.^4^ Excess histamine generated by neutrophils with chronic *P. aeruginosa* infection could contribute to the narrowing of the airways observed in asthma, allergies, chronic bronchitis, cystic fibrosis, and other lung diseases, according to Caughey.

The production of histamine by neutrophils “is a significant finding because histamine is a major player in the respiratory tract. This opens the door for new ways to look at bacterial-associated allergic inflammation,” says John Wherry, an associate professor of microbiology at the University of Pennsylvania, Philadelphia. Understanding why only certain strains induce neutrophil histamine production “could reveal insights into how pathogens evolve to evade host immunity,” Wherry says.

Caughey plans to test whether more strains of *P. aeruginosa*, other bacteria, and viruses also cause neutrophils to produce histamine. “The most common causes of severe asthma attacks are viruses,” he says. The next step would be to demonstrate that neutrophils make histamine in response to microbial infections in people. If this proves to be the case, Caughey says, “our research could improve the understanding of inflammation in bacterial infections and help us craft therapies for relief of inflammation and its consequences.”
